# Expertise development in volumetric image interpretation of radiology residents: what do longitudinal scroll data reveal?

**DOI:** 10.1007/s10459-020-09995-6

**Published:** 2020-10-08

**Authors:** Dorien van Montfort, Ellen Kok, Koen Vincken, Marieke van der Schaaf, Anouk van der Gijp, Cécile Ravesloot, Dirk Rutgers

**Affiliations:** 1 Department of Education, Utrecht University, Heidelberglaan 1, 3584CS Utrecht, The Netherlands; 2 Image Sciences Institute, Imaging Dept, University Medical Center, Utrecht, The Netherlands; 3 Center for Research and Development of Education, University Medical Center Utrecht, Utrecht, The Netherlands; 4 Department of Radiology, University Medical Center Utrecht, Utrecht, The Netherlands

**Keywords:** Radiology, Residents, Scroll patterns, Volumetric image interpretation, Visual expertise development

## Abstract

The current study used theories on expertise development (the holistic model of image perception and the information reduction hypothesis) as a starting point to identify and explore potentially relevant process measures to monitor and evaluate expertise development in radiology residency training. It is the first to examine expertise development in *volumetric* image interpretation (i.e., CT scans) within radiology residents using *scroll data* collected *longitudinally* over five years of residency training. Consistent with the holistic model of image perception, the percentage of time spent on full runs, i.e. scrolling through more than 50% of the CT-scan slices (global search), decreased within residents over residency training years. Furthermore, the percentage of time spent on question-relevant areas in the CT scans increased within residents over residency training years, consistent with the information reduction hypothesis. Second, we examined if scroll patterns can predict diagnostic accuracy. The percentage of time spent on full runs and the percentage of time spent on question-relevant areas did not predict diagnostic accuracy. Thus, although scroll patterns over training years are consistent with visual expertise theories, they could not be used as predictors of diagnostic accuracy in the current study. Therefore, the relation between scroll patterns and performance needs to be further examined, before process measures can be used to monitor and evaluate expertise development in radiology residency training.

## Introduction

The interpretation of medical images is central in radiology. Image interpretation is considered to be a highly complex task (Drew et al. 2013; Krupinski 2011; Van der Gijp et al. 2014). Errors in medical image interpretation can have significant impact on patient safety, so high-quality residency training programs are crucial for patient safety. Process measures are increasingly valued as additional sources of information about residents’ competence (Kok 2019), for example in the context of (formative) assessment and monitoring. Process measures such as computer-log data (time-stamped information on interactions with the computer, for example, scrolling, panning, windowing and zooming) and eye tracking data (time-stamped information on where a person looks, how long and in what order; Kok and Jarodzka 2016) provide information that goes beyond outcome variables, for example, about the efficiency of visual search and strategies use (Drew et al. 2013; Manning et al. 2006; Venjakob et al. 2012; Van der Gijp et al. 2017).

Whereas eye-tracking data is relatively time-consuming to collect, computer-log data can often be collected unobtrusively. Most radiological tasks are computer-based, which means that process-level data is, or can be made, available (Pecaric et al. 2017). This process-level data, in turn, could be used to monitor and assess learning and make training more adaptive. However, this requires that we know which process measures reflect developing expertise and predict performance.

In this study, we use theories on expertise development as a starting point to identify and explore potentially relevant measures. Most studies on expertise development employ cross-sectional expert-novice comparison designs (for reviews see Brams et al. 2019; Van der Gijp et al. 2017). The current study advanced previous studies on image interpretation by analysing scroll data of volumetric images collected longitudinally.

The current study had two aims. First, we investigated residents’ visual expertise development in image interpretation by examining their scroll patterns in multiple computed tomography (CT) tasks, using scroll data of CT-scan questions collected during semi-annual progress tests over five years of radiology residency training. Second, we examined whether scroll patterns were predictive for diagnostic accuracy.

## Theoretical background

### Process measures

The increasing use of digital technology in medical education and practice allows for extracting large numbers of metrics that could relate to learning and thus inform teaching and curriculum development. In radiology, studies have shown that click-level process data can provide information about the process of radiograph interpretation (Pecaric et al. 2017). CT scans additionally require learners to scroll through a large set of stacked images. Scroll movements constitute an essential human–computer interaction during volumetric image interpretation, as they normally allow observers (i.e., radiology residents) to go through the stack of slices in their own pace, and to go back and forth between slices (Den Boer et al. 2018; Van der Gijp et al. 2015). Scroll data might thus reveal how efficiently a resident interprets CT scans. Since there are many potentially relevant metrics that could be tracked in volumetric image interpretation, it is important to select specific metrics that contribute to our goal of mapping expertise development (Elias 2011). As such, theories of expertise development formed our starting point for defining these specific metrics and we cover them in the following paragraphs.

### Theories of visual expertise

Efficient visual search behaviour develops with increasing expertise as a result of acquiring an extensive and structured knowledge base. This efficient search behaviour is explained in two central theories of visual expertise (which are not mutually exclusive): the holistic model of image perception (Kundel et al. 2007) and the information-reduction hypothesis (Haider and Frensch 1999).

The holistic model of image perception states that experts are quicker to form a global impression of a radiograph than are novices. Experts’ global impressions are also more informative than those of novices and serve to guide subsequent detailed inspection of suspicious areas (Kundel et al. 2007). Support for this holistic search was found in previous studies on expertise development which revealed that expert radiologists are able to correctly identify abnormal images within approximately one-fourth of a second (e.g., Kundel et al. 2007). Likewise, experts are generally quicker to spot abnormalities than novices (Van der Gijp et al. 2017).

The information-reduction hypothesis claims that redundant and task-irrelevant information (e.g., healthy lung tissue in a chest CT scan) is strategically ignored by experts during perceptual encoding in favour of task-relevant information (Haider and Frensch 1999). For instance, in a chest CT scan the liver is usually redundant and healthy lung tissue is task-irrelevant information, while abnormal lung tissue forms task-relevant information. This was, for example, found by Manning et al. (2006), who compared the detection and localisation of pulmonary nodules in chest X-rays between radiographers and radiologists. They concluded that radiologists inspected less of the image area than radiographers due to radiologists’ ability to skip (task-irrelevant) areas.

In radiology, the holistic model of image perception and the information-reduction hypothesis are endorsed by studies that used cross-sectional eye-tracking data, 2D images (e.g., X-rays) and/or video-recorded CT-scans and found to coexist during radiological interpretation (e.g., Bertram et al. 2013; Cooper et al. 2009; Kok et al. 2012; Krupinski 1996; Kundel et al. 2007; Mallett et al. 2014). These studies have shown that experts form a global impression faster and spent more time on task-relevant areas compared to novices, which makes those measures potentially relevant measures of expertise development in volumetric images.

### Measures of expertise development in volumetric images

The high prevalence of human–computer interactions, such as scrolling and changing viewing direction in volumetric image interpretation adds to its complexity and in addition makes it more time-consuming than 2D image interpretation (Den Boer et al. 2018; Van der Gijp et al. 2015). It is not known whether findings from expertise research on 2D image interpretation, such as experts’ ability to quickly form a global impression of the image and to strategically ignore irrelevant image parts, generalize to volumetric image interpretation.

Forming a global impression of an image might be different between 2D and volumetric image interpretation (Van der Gijp et al. 2015). Whereas such a global impression can literally be formed within a single glance in 2D images (Kundel et al. 2007), for volumetric images, readers would have to ‘dive into’ the whole volumetric set of images to form an impression. Den Boer et al. (2018) and Venjakob et al. (2012) showed that this process of forming an impression is reflected in full runs: back and forward movements through more than 50% of the slices. In a think-aloud study, Den Boer et al. (2018) found that global characteristics of the image were mostly detected within the full runs, and that the analysis of these findings took place during oscillations (back-and-forth movements over smaller numbers of slices). It is thus likely that, with increasing expertise, the time needed for those full runs declines as experts are quicker in forming a global impression.

The finding that experts spend more time looking at question-relevant information compared to question-irrelevant information, defined by the information-reduction hypothesis (Haider and Frensch 1999), can easily be generalized to volumetric images: scroll data can reveal on which slices time is spent, and slices can be coded as showing task-relevant information (the abnormality) or irrelevant or redundant information. From an information-reduction hypothesis perspective, expertise could be reflected in the percentage of time spent on question-relevant areas. This variable is comparable to the variable ‘number of eye-fixations on task-relevant areas’ used in eye-tracking studies on image interpretation (e.g., Manning et al. 2006). Indeed, this metric not only reflects expertise in 2D static images, but also in videos (e.g., Balslev et al. 2012 in paediatrics, Bertram et al. 2013 in video recording of CT scans and Mallett et al. 2014 in 3D CT colonographic videos) and even in a laparoscopic surgery training (Wilson et al. 2010). Thus, the time spent on full runs and the time spent on relevant slices are considered promising measures coming from established expertise theories.

### Diagnostic accuracy

Although a high level of experience is assumed to be related to more accurate diagnoses, there is yet limited evidence that scroll-data or even eye-tracking metrics relate to observers’ diagnostic accuracy. However, such a relationship is required if those metrics are to be used for assessment or adaptive instruction (Kok 2019).

A recent study by Kelly et al. (2016) investigated the relation between eye-tracking metrics and diagnostic accuracy at different levels of visual expertise in chest radiograph interpretation. They examined the development of chest radiograph interpretation skill through medical training by measuring both eye movements and diagnostic accuracy. Their study revealed significant correlations between eye-tracking metrics (e.g., time to first eye fixation on abnormality) and the diagnostic accuracy of medical interns, 2nd-year medical residents, 4th-year radiology residents and radiologists. It is unclear whether these findings hold true for volumetric images and whether these findings generalise to scroll data, since this topic is investigated sparsely.

In sum, it needs to be further examined if scroll patterns can predict residents’ diagnostic accuracy. Specifically, if scroll patterns develop as expected based on visual expertise theories, they might be a predictor of diagnostic accuracy.

## Current study: the use of longitudinal scroll data to examine expertise development in volumetric image interpretation

The overall aim of the current study was to gain insight in radiology residents’ expertise development in volumetric image interpretation over their five-year residency program, to be able to align residency training with residents’ level of understanding in the future. To accomplish this, a data analysis was conducted on data collected between September 2013 and April 2018, including longitudinal data of all Dutch radiology residents who participated in semi-annual mandatory progress tests (The Dutch Radiology Progress Test or DRPT; Ravesloot et al. 2017).

### Study part 1: the identification of residents’ scroll patterns

The first part of our study focused on the identification of scroll patterns in CT-scans. Specifically, we investigated if residents’ longitudinally collected scroll data of CT-scan questions were in line with the holistic model of image perception and/or the information-reduction hypotheses. The study is restricted to focal diseases, that is, abnormalities that are located at a specific location where the rest of the scanned body part is relatively unaffected (Kok et al. 2012). The time spent on relevant slices is a promising measure, but this measure is only relevant for focal and not for diffuse diseases.

We hypothesized that the average percentage of time spent on full runs on a single DRPT (*PercTimeFullRunsAvg*) decreased *within* residents over time, because we expected that growing knowledge results in more informative and quicker initial impressions (Hypothesis 1a).

Furthermore, we hypothesized that the average percentage of time spent on question-relevant areas in a single DRPT (*PercTimeRelAreaAvg*) increased *within* residents over time, because we expected that a growing knowledge base improves residents’ ability to differentiate between question-relevant and question-irrelevant information (Hypothesis 1b).

### Study part 2: predicting residents’ diagnostic accuracy with residents’ scroll patterns

Subsequently, we investigated whether scroll patterns were predictive for diagnostic accuracy (*DiagAcc*), i.e. the proportion of correct answers on CT-scan questions in a DRPT. We expected a negative relation between *PercTimeFullRunsAvg* and *DiagAcc,* as it was expected that a decrease in *PercTimeFullRunsAvg* (i.e., quicker global search) on DRPTs over residency training years would be related to more accurate diagnoses (Hypothesis 2a). Furthermore, we expected a positive relation between *PercTimeRelAreaAvg* in CT-scan questions and *DiagAcc* (Hypothesis 2b), as it was expected that an increase in *PercTimeRelAreaAvg* on DRPTs over residency training years relates to more accurate diagnoses.

## Methods

### Participants

**Fig. 1:**
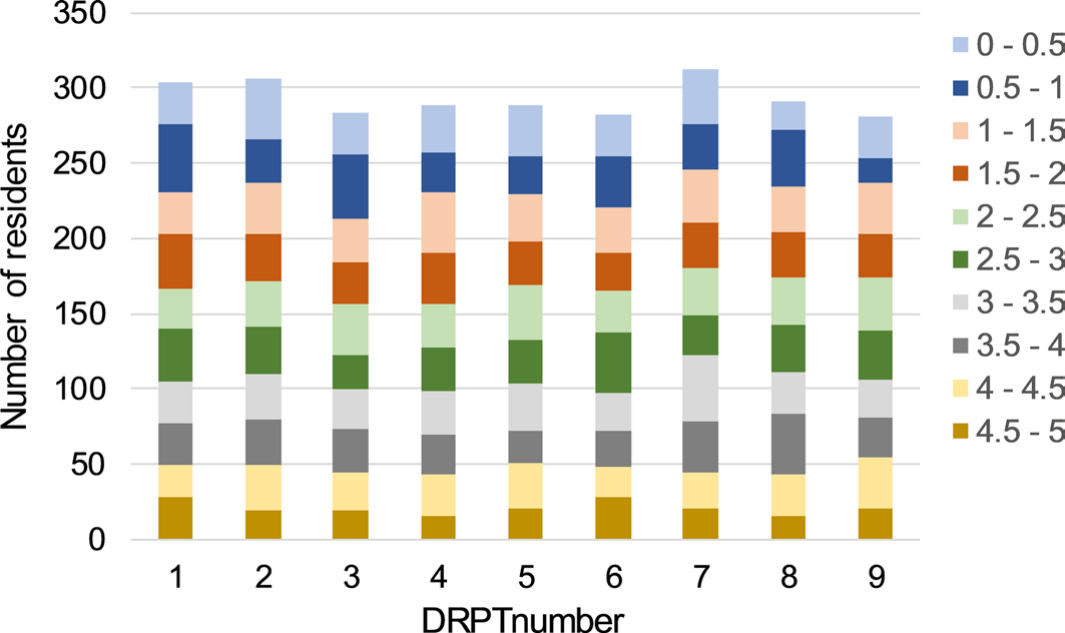
Number of residents per DRPT, categorised according to their relative training time (to control for differences in pace, training time was standardized to a five-year scale). Note that the relative time in training is a continuous factor in the dataset, but is binned here for illustration purposes

The current study used logfiles and test results of all Dutch radiology residents who participated in semi-annual DRPTs between September 2013 and April 2018, who provided permission to use their data and who had eligible data for the variable relative training time (*N*_total_ = 654 n_pertimepoint_ ± 300; see Fig. 1). Specifically, logfiles of CT-scan questions tracked the exact slice number that was depicted in the viewing direction, axial, sagittal, or coronal, at any moment. A total of 9 DRPTs was examined (the autumn 2015 test failed due to technical reasons). The number of DRPTs made per resident varied from 1 to 9 (*mean *=4.1*, SD *= 2.3), as this was dependent on residents’ years of training at the beginning of 2013 and the number of years the resident needed to complete the (five-year) residency program. For example, a resident who had completed 25 months of residency training at the beginning of 2013 and who completed the residency program in five years, participated in five DRPTs between September 2013 and April 2018.

### Instruments

#### Dutch radiology progress test

The DRPT is a semi-annual progress test that is mandatory to all Dutch radiology residents. It consists of 180 test items (up to 2013: 200 items), covering all radiology subdomains (Rutgers et al. 2018). Support for the external validity of this progress test can be found in Ravesloot et al. (2015b). As it is a progress test, all residents make the same DRPT at the same timepoint irrespective of their training stage. The test includes image questions that intend to test residents’ image interpretation skills (e.g., including CT-scans and MRI series) and non-image questions that aim to test residents’ factual radiological knowledge. Log data generated during image questions of the DRPTs, providing detailed information on how images were manipulated by residents (i.e., scroll movements and change of viewing direction over time), and test scores were collected for all DRPTs. Cronbach’s alpha for test reliability of *image questions* in DRPTs made between 2005 and 2009 varied between .87 and .91 when corrected for number of items with the Spearman Brown formula (Ravesloot et al. 2015a). In addition, Cronbach’s alpha for test reliability of *all questions* in DRPTs made between 2003 and 2016 varied between .83 and .93 (Rutgers et al. 2018). We find it reasonable to assume that image questions in DRPTs made between September 2013 and December 2018, the time period of the current study, had a similar test reliability.

#### Selection of image questions

The current study used a subset of questions per DRPT, selected on three criteria in order to obtain a homogeneous set of questions to investigate. First, only image questions that needed the interpretation of a single CT-scan were included (*n* = 83). Second, only CT-scan questions in which residents needed to provide a diagnosis were selected (leaving *n* = 78); we excluded questions that only asked to mark a normal anatomical structure representing only a part of the image interpretation task. Third, only questions with a question-relevant CT-scan volume covering *less* than 50 percent of the total provided CT-scan volume were included (leaving *n* = 48). The third criterium ensured the inclusion of only focal diseases (localized abnormalities) and the exclusion of CT-scan questions concerning diffuse diseases (distributed abnormalities). From a theoretical perspective, Kok et al. (2012) showed that diffuse diseases evoke different visual search strategies than focal diseases. Therefore, it is better to discriminate between these types of diseases in image interpretation studies. From a methodological perspective, variation in the variable *PercTimeRelArea* (i.e., percentage of time spent on question-relevant areas) could be too low for questions with a high coverage rate.

In total, 48 CT-scan questions were selected with an average of five questions per DRPT (min = 2, max = 9). A more detailed description of the question-selection process can be found in “Appendix 1”.

#### Selected image questions

The included CT-scan questions (n = 48) cover six radiology subdomains (see Table 1). Three question formats can be distinguished. First, marker questions (n = 2) in which residents needed to flag an abnormality that was in correspondence with a provided diagnosis (e.g., “Place the marker in the subarachnoid haemorrhage within the interpeduncular cistern”). Second, multiple choice questions (n = 32) in which residents either needed to select the most likely diagnosis from a given set of diagnoses or needed to indicate if a provided statement was correct (e.g., “the round lesion in the right lower lobe likely indicates a round atelectasis”). Third, long list menu questions (n = 14) in which residents needed to select the most likely diagnosis in a drop-down menu displaying 1000 + possibilities.

**Table 1:** Number of questions selected per radiology subdomain and medical case

Radiology subdomain	Case								Total
	Normal anatomy	Anatomical variant	Acute disease	Chronic disease	Mass	Trauma	Vascular disease	Congenital disease	
Cardiovascular and thoracic		4			6	2	1		13
Neuro and head-and-neck		1		1	4	2	6		14
Abdominal	2		4	3	5	1	1	1	17
Child					1				1
Intervention							1		1
Musculoskeletal				1	1				2
Total	2	5	4	5	17	5	9	1	48

### Procedure

DRPTs were taken in a computer hall at a university in the Netherlands, using digital test software VQuest (“Volume Quest”, http://www.vquest.eu) that is found to be valid, accurate, and user-friendly (Ravesloot et al. 2015b; Rutgers et al. 2018). Questions were displayed page-by-page and residents were able to navigate back and forth between questions. Informed consent for collecting and using logfile data was obtained from all individual participants included in the study via a digital form at the start of each DRPT.

**Fig. 2:**
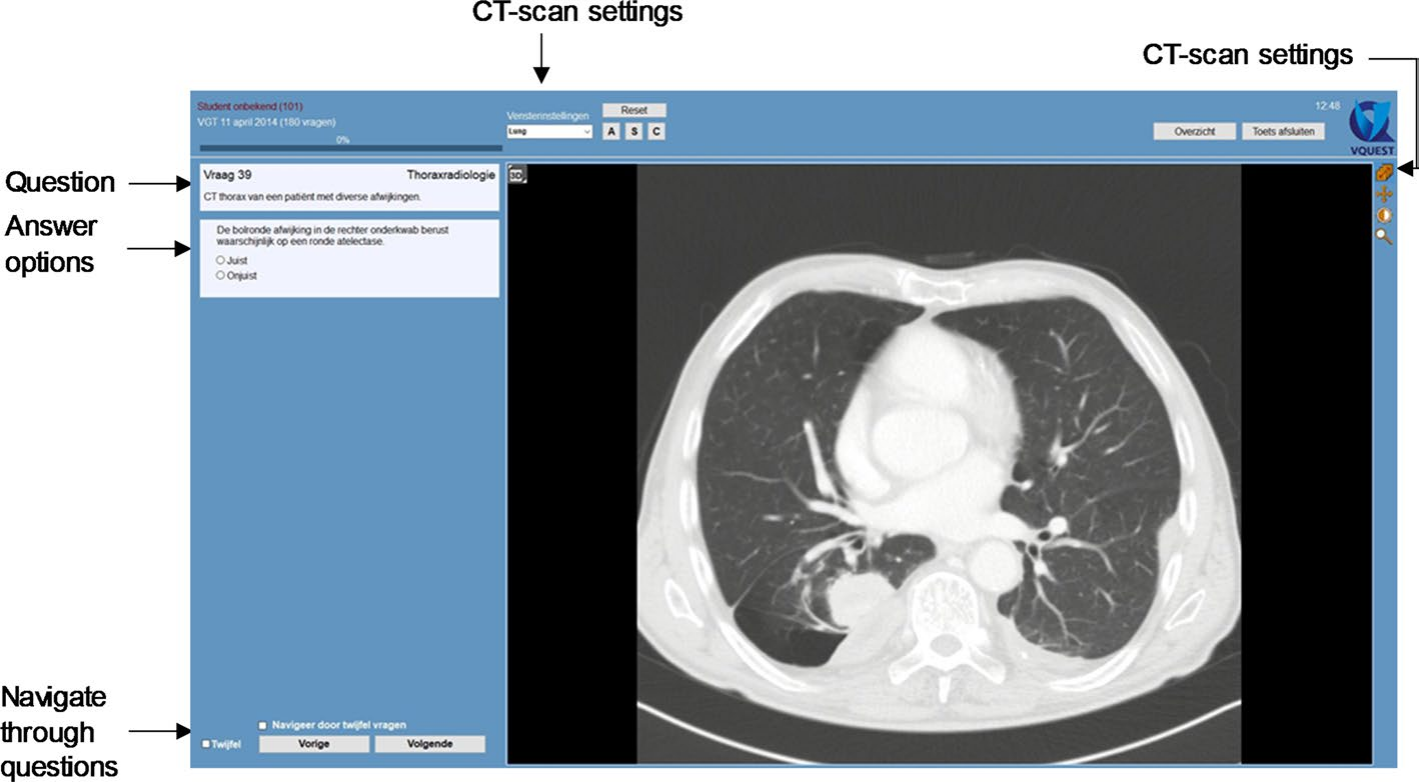
Example of a CT-scan question as displayed to residents in a DRPT

CT scans of image questions were displayed in gray scale and were presented on a 15.6-inch conventional computer monitor with a 1366 × 768 pixel resolution. In all CT-scan questions, residents were allowed to scroll through the stack of images, to pan the image, to change window-level settings and to zoom in and out. Moreover, CT images could either be viewed in sagittal, coronal, and axial viewing direction (x, y, z; 35 out of 48 questions), or only in axial viewing direction (z; 13 out of 48 questions). Figure 2 shows a typical example of a CT-scan question as displayed to residents in a DRPT.

#### Residents’ training time

Not all residents went straight through the five-year residency program, because of, for example, part-time work or research engagement. To correct for differences in pace, training time (*TrTime*) was standardized to a five-year scale at the time of each test (see Eq. 1). For six residents we were unable to derive the date of training enrolment (*StartDate*), these were excluded from all analyses (leaving n_total_ = 648). *TrTime* was treated as a continuous variable; residents made multiple DRPTs over time (varying from 1 to 9 measures) and the variable contained all possible time points in months between zero and five relative training years due to the fact that *DRPTdates* were not connected to a specific training stage.

1 $$TrTime = \left( {\frac{DRPTdate - StartDate}{EndDate - StartDate}} \right)*5.$$

#### Percentage of time spent on full runs

**Fig. 3:**
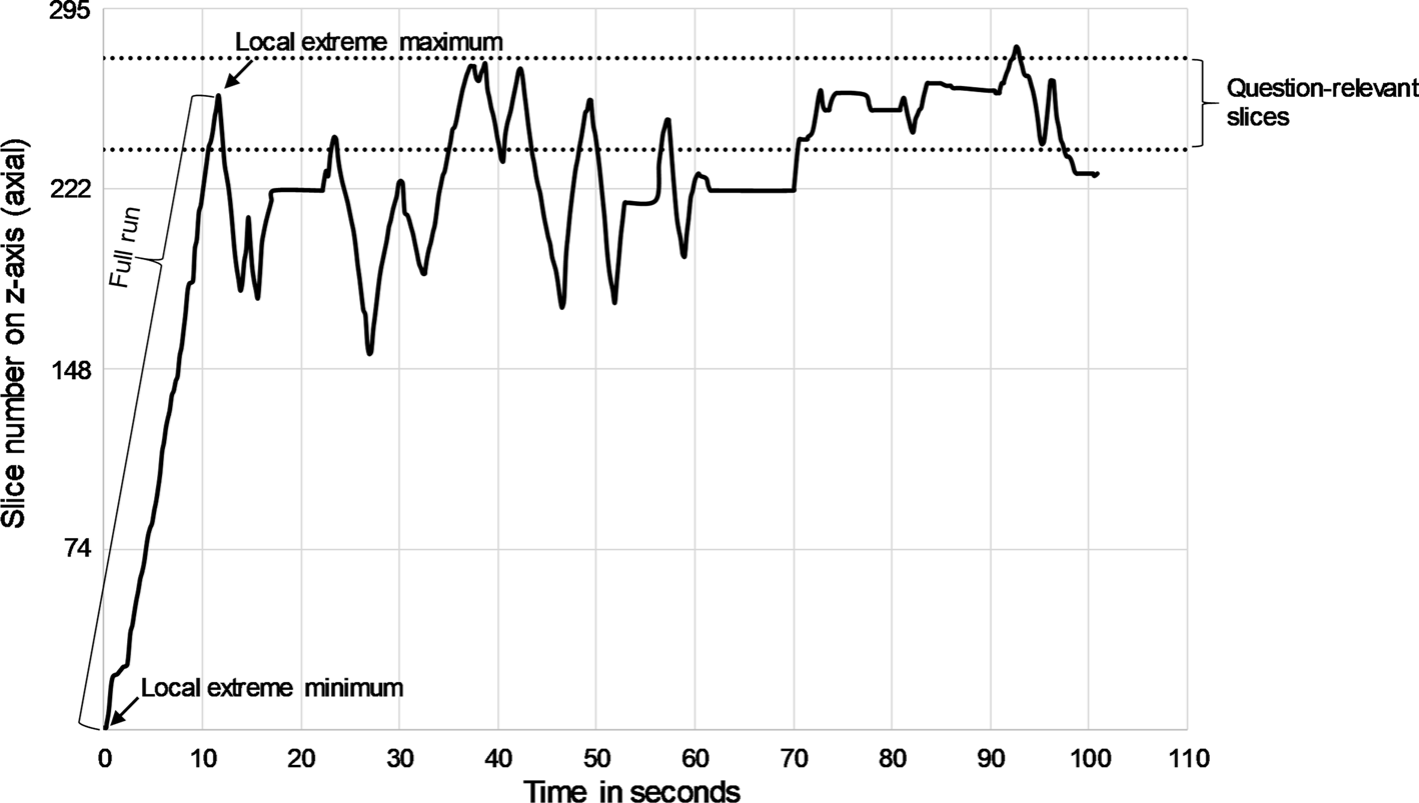
Graphical representation of a logfile of a CT-scan question made by a resident. Note that this question was viewed only in axial direction by the resident

Figure 3 shows a graphical representation of a logfile of a CT-scan question made by a resident, to indicate how the *PercTimeFullRuns* (scroll movements through more than 50% of the slices) was derived from the log data. First, we identified full runs in each CT-scan question in a similar manner as Den Boer et al. (2018). Specifically, we calculated the difference in slice number between each local extreme minimum and a previous local extreme maximum (and vice versa). In case the difference between two extremes (i.e., slice numbers on z-axis) made up more than 50% of the slices, the run was viewed as a full run. The *PercTumeFullRuns* was the duration of all full runs in milliseconds divided by the total amount of time spent on that question.

#### Percentage of time spent on question-relevant areas

To derive the *PercTimeRelArea*, percentage of time spent on question-relevant areas by residents in each CT-scan question, three expert radiologists of the radiology department of UMC Utrecht (DR, AG, CR) determined the question-relevant CT-scan area of each question. See "Appendix 1" for more information on how the question-relevant area was determined. Subsequently, we continuously compared residents’ scroll position (in slice number in x, y or z direction) of the current viewing direction (default z-axis; axial view) to the question-relevant slice numbers on that axis. In case a resident viewed a question-relevant slice number the time was logged (see for a graphical example Fig. 3). Finally, we divided the total time spent on question-relevant areas by the total time spent on each question to derive the *PercTimeRelArea*.

#### Diagnostic accuracy

Residents received a score of 1 for correct answers and a score of 0 for incorrect answers. These scores were used as an indicator of their diagnostic accuracy (*DiagAcc*).

#### Dutch radiology progress test difficulty

As an indicator of DRPT difficulty (*DRPTdiff*), we calculated the average difficulty of the selected questions per DRPT. Question difficulty was defined by the proportion of correct answers on that question, also known as the *P* value. *DRPTdiff* was rescaled on a scale from − 0.5 to 0.5: a value of 0 corresponded to a DRPT with an average question difficulty of 0 (i.e., 50 percent of the residents provided the correct answer). Since the *DRPTdiff* was based on the proportion correct, a higher score for *DRPTdiff* indicates a less difficult DRPT.

### Data analysis

The data used had a very specific structure. Not only were questions nested within DRPTs within residents, questions also differed per DRPT, residents made different DRPTs at a certain training stage (i.e., *TrTime*) and participated in a different number of DRPTs (i.e., dependent on residents’ *StartDate*). At first glance, the nesting suggested the use of longitudinal multilevel models with the item level as the lowest level of analysis. However, due to the specific data wave structure this was not possible. Specifically, while checking the necessity of the second level (DRPT level) in a three-level model, we would test the interdependency hypothesis stating that ‘residents values on the *outcome* variable (e.g., *PercTimeFullRuns* and *PercTimeRelArea*) are more alike on questions within DRPTs than on questions between DRPTs’. When comparing values on the outcome variable between DRPTs we are in fact comparing residents to themselves (i.e., residents made multiple DRPTs) and to others (i.e., residents only made a set of DRPTs) *at the same time*, making the model invalid. We illustrate this with a part of the dataset: 304 residents participated in the first DRPT and 306 residents participated in the second DRPT of which 237 residents also participated in the first DRPT. From this it follows that 69 residents in the second DRPT were new in the dataset and 67 residents of the first DRPT left the dataset. Consequently, comparisons on the outcome variable between the first and the second DRPT would be based on comparisons within residents over DRPTs *and* between residents *simultaneously*, resulting in an invalid model. Hence, we needed to aggregate all data (i.e., scroll data and data of test results) on the DRPT level and use this level as the lowest level of analysis in all analyses. We therefore also corrected for differences in difficulty on the DRPT level (*DRPTdiff*) in all analyses, instead of correcting for differences in question difficulty. In order not to neglect the item level, we conducted explorative analyses on the item level using descriptive statistics.

#### Study part 1: the identification of residents’ scroll patterns

Before examining if the average percentage of time spent on *full runs* (*PercTimeFullRunsAvg*) decreases within residents over time (H1a) and if the average percentage of time spent on *question*-*relevant areas* (*PercTimeRelAreaAvg*) increases within residents over time (H1b), we inspected the data visually on the item level using IBM SPSS Statistics Version 25. Specifically, we plotted the dependent variables *PercTimeFullRuns* and *PercTimeRelArea* against the predictor *TrTime* for each selected CT-scan question. Hence, these plots were cross-sectional; displaying all data of residents on one CT-scan question on one DRPT. These plots were used to inspect if all questions showed roughly the same data pattern, in order to determine if aggregation on the DRPT level was appropriate. In addition, we fitted a linear line in all plots to see if expected relations between *TrTime* and the dependent variables *PercTimeFullRuns* (i.e., decrease over *TrTime*) and *PercTimeRelArea* (i.e., increase over *TrTime*) were visible cross-sectionally.

Second, after assumption checks, we performed two two-level longitudinal regression analyses with the dependent variables *PercTimeFullRunsAvg* (H1a) and *PercTimeRelAreaAvg* (H1b). The first level represented the DRPT level (measurements within residents) and the second level the resident level (measurements between residents). For the analysis of each aggregated dependent variable, we ran multiple successive models in HLM6 to determine the best fitting model (in line with the longitudinal multilevel-analysis strategy described in Hox et al. 2018): Step by step we extended the intercept only model that included a fixed effect for *TrTime* and random intercepts on the resident and the DRPT level (Model 1), with a fixed effect for *DRPTdiff* (Model 2) and with a random slope for *TrTime* (Model 3). We used full maximum likelihood estimation in all analyses. Only regression coefficients of the best fitting models were interpreted. Model equations of the best fitting models are displayed in “Appendix 3”.

#### Study part 2: predicting residents’ diagnostic accuracy with residents’ scroll patterns

Before examining if the *PercTimeFullRunsAvg* and *PercTimeRelAreaAvg* are predictive for *DiagAcc* (H2a and H2b), we inspected the data visually on the item level using IBM SPSS Statistics Version 25. Similar to Study Part 1, we plotted the *PercTimeFullRuns* and *PercTimeRelArea* against the *TrTime* per CT-scan question. This time, however, we set markers by *DiagAcc* (correct versus incorrect). These cross-sectional plots were used to inspect if all questions displayed roughly the same data pattern regarding *DiagAcc* in specific, in order to determine if aggregation on the DRPT level was appropriate.

Second, we performed a two-level longitudinal logistic regression analysis, using the number of questions correct per number of (for the current study selected) questions in a DRPT as outcome variable. Note that *DiagAcc* (i.e., correct versus incorrect answers) is aggregated on the DRPT level by calculating the number of correct answers per DRPT for each resident and the total number of questions made per DRPT. Similar to the first part of the study, the first level represented the DRPT level (measurements within residents) and the second level the resident level (measurements between residents). Furthermore, in order to deal with the proportional outcome variable, we used a logit-link function on the first level. Specifically, we performed a logit transformation to be able to scale the outcome variable, the first level variance (the resident level) was fixed (cf. Hox et al. 2018). We ran multiple successive models in SuperMix Version 1.10 to determine the best fitting model in line with the general analysis strategy described in Sommet and Morselli (2017). Step by step we extended the intercept only model that included a fixed effect for *TrTime* and a random intercept on the DRPT level, with fixed effects for *DRPTdiff, PercTimeFullRunsAvg* and *PercTimeRelAreaAvg*, and with a random slope for *TrTime*. We chose SuperMix as it provides the possibility to use adaptive quadrature estimation (i.e., number of quadrature points is set to 20), an estimation procedure that is more accurate than the penalized-quasi likelihood estimation which is available in HLM6 (Rabe-Hesketh et al. 2005). Only regression coefficients and odds ratios of the best fitting model were interpreted. Model equations of the best fitting models are displayed in Appendix 3.

#### Significance level, outliers, missing values, and the calculation of the explained variance

For all statistical analyses, in both Study Part 1 and 2, a significance level of α = .05 was used as boundary of significance. For all dependent and independent variable combinations we visually inspected question-level (cross-sectional) plots for outliers. As there were no clear outliers on the item level, we did not exclude any cases of the data aggregated on the DRPT level. Furthermore, due to technical failure nine logfiles of random resident and question combinations were missing in the item level dataset, since this number is negligible in regard to the size of the dataset we treated these missing values as missing listwise in all analyses.

As measure of effect size we reported the explained variance (R^2^) on the first level (within residents) of the best fitting multilevel models when compared to their corresponding unconditional means models with no predictors at the first and second level (i.e., reduction of unexplained variance; e.g., Hox et al. 2018; LaHuis et al. 2014). While the unconditional means model in longitudinal multilevel analyses generally overestimates the within level variance (first level) and underestimates the between level variance (second level) (e.g., Hox et al. 2018), our main interest was in the variable *TrTime*. Comparison with the intercept only models was the only way to provide an indication of the explained variance by the variable *TrTime* in each dependent variable, that is *PercTimeFullRunsAvg* and *PercTimeRelAreaAvg*.

## Results

### Study part 1: the identification of residents’ scroll patterns

#### Scroll patterns on the item level

**Fig. 4:**
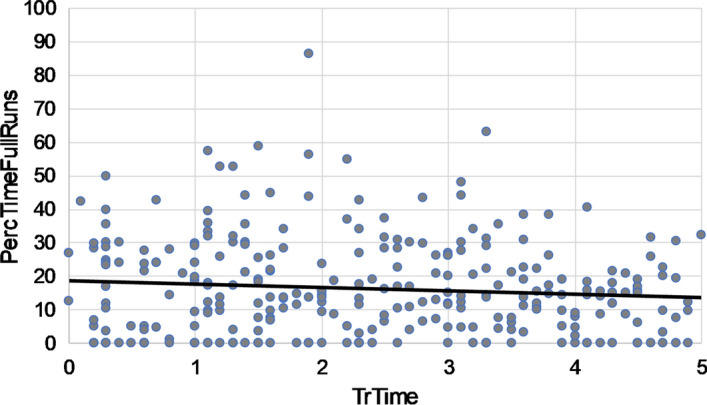
Example of a cross-sectional scatterplot of the percentage of time spent on fullruns (PercTimeFullRuns) against residents relative training time (TrTime) on a specific CT-scan question. This question was used as example, since the slope of the linear fitted trendline (i.e., − 0.92) was closest to the average of the slopes of trendlines in the cross-sectionplots of all 48 questions (i.e., − 0.81)

**Fig. 5:**
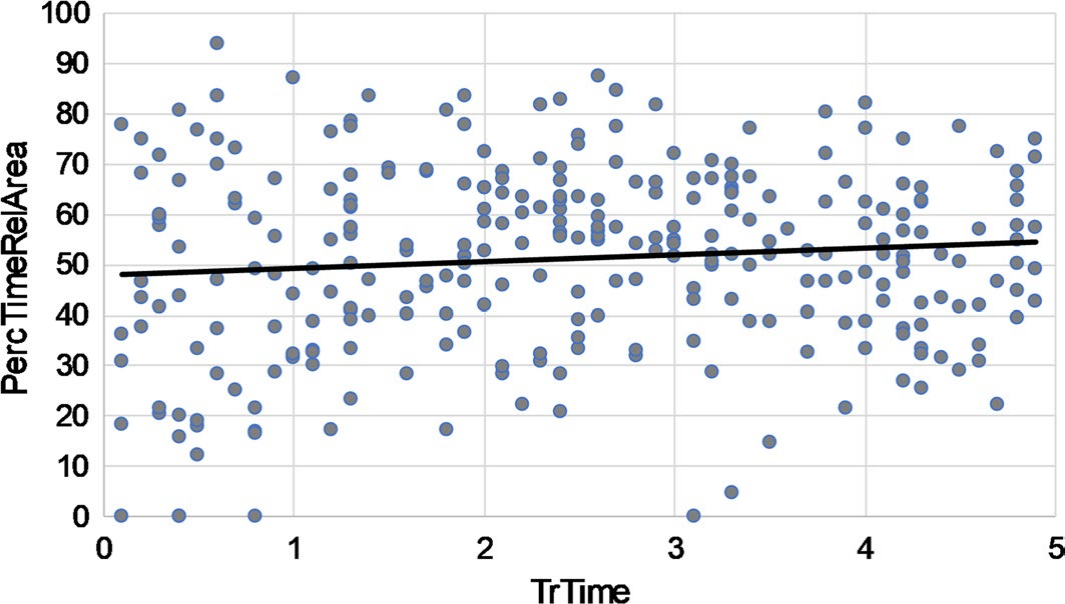
Example of a cross-sectional scatterplot of the percentage of time spent on relevant area (PercTimeRelArea) against residents relative training time (TrTime) on a specific CT-scan question. This question was used as example, since the slope of the linear fitted trendline (i.e., 1.19) was closest to the average of the slopes of trendlines in the cross-sectionplots of all 48 questions (i.e., 1.32)

Cross-sectional scatterplots of *PercTimeFullRuns* and *PercTimeRelArea* against *TrTime* did not reveal differences in data patterns between CT-scan questions: neither for specific radiological subdomains nor for question format. Overall, in cross-sectional plots of *PercTimeFullRuns* against *TrTime* (see Fig. 4), the slope parameter of the fitted linear trend line suggested a negative relation in 36 out of 48 questions: Residents that were further in the training program spent a lower percentage of time on full runs (i.e., the holistic phase of image perception). In addition, in cross-sectional plots of *PercTimeRelArea* against *TrTime* (see Fig. 5), the slope parameter of the fitted linear trend line suggested a positive relation in 41 out of 48 questions: Residents that were further in the training program spent a higher percentage of time on question-relevant areas. These findings should, however, be interpreted with caution, as we did not perform statistical analyses.

#### Scroll patterns on the DRPT level

Descriptive statistics of the dependent variables *PercTimeFullRunsAvg* and *PercTimeRelAreaAvg* are displayed in Table 2. DRPTs had an average question difficulty of 0.14 (min = 0.02, max = 0.24, *SD *= 0.07; i.e., 64 percent of the residents provided the correct answer).

**Table 2:** Descriptive statistics of residents’ PercTimeFullRuns and PercTimeRelArea Averaged on DRPT level by Relative Time in Training

Relative time in training	*n*^a^	PercTimeFullRuns averaged per DRPT		PercTimeRelArea	
				averaged per DRPT	
		Mean	SD	Mean	SD
0–0.5	271	20.3	10.5	43.0	14.1
0.5–1	287	20.9	9.9	46.3	13.2
1–1.5	293	19.4	9.7	49.4	12.8
1.5–2	272	18.6	9.7	49.6	12.0
2–2.5	282	18.4	9.3	49.1	12.2
2.5–3	276	18.5	9.7	51.9	11.4
3–3.5	270	17.8	9.7	50.3	12.0
3.5–4	258	18.2	9.1	51.9	12.6
4–4.5	239	16.6	9.1	50.7	11.6
4.5–5	188	17.1	9.3	52.0	12.2

Whereas relative time in training (TrTime) was used as a continuous factor in all analyses, it is binned here for illustration purposes

^a^Some residents are more than once present in one relative training time interval since they made more than one DRPT within the binned interval

For both two-level longitudinal regression analyses (i.e., dependent variables *PercTimeFullRunsAvg* and *PercTimeRelAreaAvg*) the assumptions of linearity and the absence of multicollinearity were met. The assumption of normality of first level residuals was sometimes slightly violated, which is why all reported results are based on estimations with robust standard errors (cf. Dedrick et al. 2009; Hox et al. 2018). In addition, the assumption of homoscedasticity was violated to a minor degree. In line with Goldstein (1999), we modelled the first level variance as function of the predictor *TrTime* (i.e., heterogeneous variances) in an attempt to minimise the violation of homoscedasticity. This, however, did not solve the issue. Furthermore, since there was no theoretical basis to model the variance as function of the predictor *TrTime*, all reported results are based on the initial models described in the method section: models with fixed effects for the independent variables that are examined and random intercepts on the resident and the DRPT level.

#### Average time spent on full runs per DRPT

For the two-level longitudinal model with the dependent variable *PercTimeFullRunsAvg*, a model that included fixed effects for *TrTime* and *DRPTdiff* as well as random intercepts on DRPT and resident level (Model 2) was the best fitting model. Model results are displayed in Appendix 2 and Table 3. The explained variance on the first level by the predictors *TrTime* and *DRPTdiff* is 0.03, based on a comparison with the intercept only model as explained in the Method section. This indicates that only three percent of the variation in the percentage of time spent on full runs per DRPT *within* residents can be explained by *TrTime* and *DRPTdiff*.

**Table 3:** Results of multilevel analysis; outcome variable PercTimeFullrunsAvg

Predictors	M_2_: TrTime + DRPTdiff		
Fixed effects	*b*	*SE*	*p*
Intercept	20.19	0.52	< .001
TrTime	− 0.94	0.14	< .001
DRPTdiff^a^	6.70	2.35	.005

Random effects	Vcomp	*SD*	*p*
σ_e_^2^	81.56	9.03	
σ_µ0_^2^	10.84	3.29	< .001
Deviance	19,350.0		

*b *= coefficient; *SE *= Standard error; *p *= *p*-value; Vcomp = Variance component

*SD *= Standard deviation

^a^Measured on a scale from − 0.5 to 0.5

The intercept of 20.2 of Model 2 refers to the average percentage of time spent on full runs on a DRPT with an average question difficulty of 0 (i.e., 50 percent of the residents provided the correct answer), of residents who are at the start of the five-year residency training program. Regarding H1a, a significant negative linear relation between *TrTime* and *PercTimeFullRunsAvg* was found (*b *= − 0.94), indicating that moving one year forward in the radiology training program led to a decrease of the percentage of time spent on full runs per DRPT of 0.94 percent point. The absolute decrease in time spent on full runs depended on both the total time spent on CT-scan questions within a certain DRPT and the number of questions within that DRPT, since DRPTs varied within and between residents we could not calculate this absolute decrease in time. We do know, however, that residents spent on average 122.9 s (*SD* = 124.1) on a CT-scan question. Therefore, regarding H1a, the absolute decrease in time spent on full runs when moving one year forward in the radiology training program was most likely in the range of a few seconds per DRPT. A significant positive linear relation between *DRPTdiff* and *PercTimeFullRunsAvg* was found (*b *= 6.70). In terms of the overall intercept an increase of the average question difficulty on a DRPT towards 0.1 (i.e., 60 percent of the residents provided the correct answer), led on average to an increase of 0.67 percent point in the percentage of time spent on full runs. This indicates an increase in residents’ global search; the holistic phase of image perception, on that DRPT. Note that the observed *DRPTdiff* ranged between 0.02 and 0.24, therefore a maximum increase in the percentage of time spent on question-relevant areas on a DRPT in light of the current data is 1.6 percent point. Moreover, the absolute increase in percentage of time spent on full runs per DRPT was most likely in the range of a few seconds per increase of .1 in *DRPTdiff*.

#### Average time spent on question-relevant areas per DRPT

For the two-level longitudinal model with the dependent variable *PercTimeRelAreaAvg*, a model that included fixed effects for *TrTime* and *DRPTdiff* as well as random intercepts on the DRPT and the resident level (Model 2), was the best fitting model. Model results are displayed in “Appendix 2” and Table 4. The explained variance on the first level by the predictors *TrTime* and *DRPTdiff* is 0.17, based on a comparison with the intercept only model as explained in the Method section. This indicates that 17 percent of the variation in the percentage of time spent on question-relevant areas per DRPT *within* residents can be explained by *TrTime* and *DRPTdiff*.

**Table 4:** Results of multilevel analysis; outcome variable PercTimeRelAreaAvg

Predictors	M_2_: TrTime + DRPTdiff		
Fixed effects	*b*	*SE*	*p*
Intercept	53.16	0.64	< .001
TrTime	1.70	0.18	< .001
DRPTdiff^a^	− 57.47	2.74	< .001

Random effects	Vcomp	*SD*	*p*
σ_e_^2^	133.04	11.53	
σ_µ0_^2^	5.30	2.30	.008
Deviance	20,467.9		

*b *= coefficient; *SE *= Standard error; *p *= *p* value; Vcomp = Variance component

*SD *= Standard deviation

^a^Measured on a scale from − 0.5 to 0.5

The intercept of 53.1 of Model 2 refers to the average percentage of time spent on question-relevant areas on a DRPT with an average question difficulty of 0 (i.e., 50 percent of the residents provided the correct answer), for residents that are at the start of the five-year residency training program. Regarding H1b, a significant positive linear relation between *TrTime* and *PercTimeRelAreaAvg* was found (*b *= 1.7), indicating that moving one year forward in the radiology training program led on average to an increase of the percentage of time spent on question-relevant areas per DRPT of 1.7 percent point. Similar to the *PercTimeFullRunsAvg* this change was most likely in the absolute time range of a few seconds per DRPT. A significant negative linear relation between *DRPTdiff* and *PercTimeRelAreaAvg* was found (b = − 57.5). In terms of the overall intercept, this indicates that an increase of the average question difficulty on a DRPT towards 0.1 (i.e., 60 percent of the residents provided the correct answer), led on average to a decrease of 5.8 percent point in the percentage of time spent on question-relevant areas on that DRPT. Note that the observed *DRPTdiff* ranged between 0.02 and 0.24, therefore a maximum decrease in the percentage of time spent on question-relevant areas on a DRPT in light of the current data is 13.8 percent point. Moreover, the absolute decrease in time spent on question-relevant areas per DRPT was most likely in the range of 10 s per increase of .1 in *DRPTdiff*.

### Study part 2: predicting residents’ diagnostic accuracy with residents’ scroll patterns

#### Predicting diagnostic accuracy on the item level

Cross-sectional scatterplots of *PercTimeFullRuns* against *TrTime* did not reveal differences in the spread of data points that were marked as either correct or incorrect (i.e., diagnostic accuracy) between CT-scan questions: neither for specific radiological subdomains nor for question format. The cross-sectional plots of the *PercTimeRelArea* against *TrTime*, on the contrary, revealed a difference in the spread of data points that were marked as either correct or incorrect between marker questions (*n* = 2), in which residents need to flag an abnormality, and other question formats (i.e., multiple choice and long list menu questions, *n* = 46). Specifically, marker questions showed a strong division between residents who provided the correct answer and residents who provided an incorrect answer: The former clearly spent more time on question-relevant area (see Table 5). Such a strong distinction was absent in plots of multiple choice questions, in which residents needed to select the most likely diagnosis from a given set of diagnoses, and in plots of long list menu questions, in which residents needed to select the most likely diagnosis out of 1000 + possibilities (see Table 5).

**Table 5:** Descriptive statistics of residents’ diagnostic accuracy on question-level

		Marker		Multiple choice		Long list menu	
		Correct	Incorrect *n* = 568	Correct	Incorrect	Correct	Incorrect *n* = 1643
		*n* = 291		*n* = 6053	*n* = 3262	*n* = 2111	
PercTimeRelArea							
Mean		19.87	5.38	48.34	46.98	58.91	58.50
SD		16.62	6.87	22.90	25.56	28.17	31.17
PercTimeFullRuns							
Mean		17.59	19.84	20.89	21.04	14.23	16.78
SD		17.27	16.41	18.58	18.66	15.45	15.07

#### Predicting diagnostic accuracy on the DRPT level

The relation between *PercTimeRelArea* and *DiagAcc* differed between marker questions and the other question formats. Unfortunately, we were not able to run a separate analysis for the marker questions, since there were only two marker questions in the dataset. Therefore, we excluded marker questions when aggregating the data on the DRPT level, in order to perform two-level logistic regression analyses with as binary outcome the number of questions correct per number of questions in a DRPT. DRPTs excluding marker questions had an average question difficulty of 0.13 (min = 0.03, max = 0.24, *SD *= 0.07; i.e., 63 percent of the residents provided the correct answer). For the two-level longitudinal logistic regression analyses the assumptions of normality of second level residuals and the absence of multicollinearity were met.

#### Diagnostic accuracy aggregated on the DRPT level

A two-level longitudinal logistic regression model that included fixed effects for *TrTime*, *DRPTdiff*, *PercTimeFullRunsAvg* and *PercTimeRelAreaAvg* as well as a random intercept on the DRPT level (Model 2), was the best fitting model that included all variables of interest. The results of Model 2 are displayed in Appendix 2 and Table 6.

**Table 6:** Results of multilevel analysis; outcome variable number of questions correct per number of questions on a DRPT

	M_2_: TrTime + PercTimFullRunsAvg + PercTimRelAreaAvg + DRPTdiff				
Fixed effects	b	SE	*p*	OR	95% CI
Intercept	− 1.078	0.14	< .001	0.34	[0.26, 0.45]
TrTime	0.338	0.02	< .001	1.40	[1.36, 1.44]
PercTimeFullRunsAvg	< .001	0.00	.833	1.00	[1.00, 1.00]
PercTimeRelAreaAvg	0.003	0.00	.067	1.00	[–, 1.01]
DRPTdiff^a^	5.093	0.34	< .001	162.92	[83.64, 317.36]
Random effects	Vcomp	*SE*	*p*		
σ_*µ*0_^2^	0.056	0.015	< .001		
Deviance	16,757.3				

*b *= regression coefficient (logit(NumberCorrect)); *SE *= Standard error; *p *= p-value; *OR *= Odds Ratio; CI = confidence interval for odds ratio (OR)

^a^ Measured on a scale from − 0.5 to 0.5

The intercept of − 1.08 of Model 2, indicates that on average the proportion of questions estimated to be correct is 0.25 for residents on a DRPT with an average question difficulty of 0 (i.e., 50 percent of the residents provided the correct answer) at the start of the five-year residency training program and with a percentage time spent on relevant areas of zero. Regarding H2a and H2b, the odds ratios of the fixed effects for *PercTimeFullRunsAvg and PercTimeFullRunsAvg* were not significantly different from 1, indicating that both the percentage of time spent on full runs and the percentage of time spent on relevant area did not significantly predict the number of questions correct per number of questions in a DRPT (i.e., diagnostic accuracy; H2a, OR = 1.001; H2b; *OR *= 1.003). There was a significant positive relation between *TrTime* and the number of questions correct per number of questions in a DRPT (i.e., diagnostic accuracy), *OR *= 1.40. This indicates that residents were 1.40 times more likely to have a larger proportion of questions correct on a DRPT if they moved one year forward in the training program. There was a significant positive relation between *DRPTdiff* and *DiaggAcc, OR *= 162.92. Indicating that in easier DRPTs residents were more likely to have a larger proportion of questions correct. This result is not surprising, since *DRPTdiff* represented the average percentage of answers correct on questions within a DRPT of all residents.

## Discussion

In this study, we used theories on expertise development as a starting point to identify and explore potentially relevant measures to monitor and evaluate expertise development in radiology, to eventually improve radiology training programs. Prior work on expertise development in medical image interpretation was mainly based on cross-sectional eye-tracking data in 2D images, like X-rays (e.g., Kok et al. 2012; Krupinski 1996; Kundel et al. 2007; Manning et al. 2006). The current study examined radiology residents’ development of expertise in *volumetric* image interpretation (i.e., CT-scans) using *scroll data* collected *longitudinally* over five years of residency training. First, we investigated if residents’ scroll patterns align with the holistic model of image perception and the information reduction hypothesis. Second, we examined if scroll patterns can predict diagnostic accuracy.

In line with H1a, we found that the average percentage of time spent on full runs by residents on a DRPT (i.e., holistic phase of image perception) decreased within residents as they moved forward in the training program. Two potential causes of the decrease in the percentage of time spent on full runs are: (1) the absolute time spent on full runs stays the same over training years, but the total time spent on questions increases, resulting in a decrease in the percentage of time spent on full runs and (2) the absolute time spent on full runs decreases over training years (i.e., quicker holistic phase with increasing levels of expertise), whereas the time spent on questions stays the same, leading to a decrease in the percentage of time spent on full runs. Additional cross-sectional scatterplots on the item level, i.e., absolute time spent on full runs against relative training time and total time on question against relative training time, suggested that residents did *not* spend more time on questions when they are further in the training program. The scatterplots, however, did suggest that residents spent less (absolute) time on full runs when they are further in the training program for most questions (41 out of 48). Therefore, a decrease in the percentage of time spent on full runs is most likely a consequence of a decrease in time spent on global search (i.e., the holistic phase). This finding extends results from cross-sectional eye-tracking studies that showed that the holistic model of image perception is reflected in observers’ eye movements while inspecting 2D images or video-recorded CT-scans (e.g., Cooper et al. 2009; Krupinski 1996; Mallett et al. 2014).

In line with H1b, we found that the average percentage of time spent on question-relevant areas by residents on a DRPT increased *within* residents as they moved forward in the training program. Therefore, we conclude that residents’ scroll patterns are in line with the information reduction hypothesis: More experienced residents take in more task-relevant information and less task-irrelevant information as a consequence of their growing ability to differentiate between task-relevant and task-irrelevant information. This finding extends findings from cross-sectional eye-tracking studies that showed that the information reduction hypothesis is reflected in observers’ eye movements (e.g., Bertram et al. 2013; Krupinski 1996; Manning et al. 2006).

The absolute changes in scroll patterns over time are relatively modest: Each training year results in the holistic impression taking a few seconds of time less, whereas the time spent on question-relevant information increases with a few seconds per DRPT. However, considering cross-sectional eye-tracking studies (e.g., Krupiniski 1996; Mallet et al. 2014), the absolute time differences between training years, found in the current study, are comparably quite large. This is probably due to the nature of scroll data: scroll movements physically take time, eye-movements can be executed much faster. Furthermore, a large proportion of the variation in the scroll data (i.e., *PercTimeFullRunsAvg* and *PercTimeRelAreaAvg*) could not be explained by the variables in the current dataset. Although not directly visible in question-level cross-sectional plots, potential factors influencing scroll patterns could have been differences in strategy use for different question formats, the number of slices present in the scan, abnormality, and/or radiological subdomain. For example, in multiple choice questions residents might use an exclusion strategy, i.e. excluding the provided diagnosis one-by-one in order to end up with the correct diagnosis. By doing so they will spent quite some time on question-irrelevant areas.

In the second part of the study we examined if scroll patterns can predict diagnostic accuracy. Contrary to H2a, we found that the percentage of time spent on full runs by residents on a DRPT (i.e., global search) did *not* significantly predict diagnostic accuracy. Hence, the percentage of time spent on full runs cannot be used as a predictor for diagnostic accuracy. An explanation for this could be that forming a faster global impression is only beneficial when observers are time-pressured: Being quicker in the holistic phase leaves more time to scrutinize image details. As time pressure is an aspect of daily radiology practice, it is interesting to investigate its influence further in future studies.

In addition, contrary to H2b, we found that the percentage of time spent on question-relevant areas by residents per DRPT did not significantly predict diagnostic accuracy. Hence, the percentage of time spent on question-relevant areas cannot be used as a predictor for diagnostic accuracy. A reason for this might be that—in order to be able to provide the correct diagnosis—residents not only need to know where to look, but also need to characterise findings and generate a differential diagnosis in order to provide the correct diagnosis (cf. Crowley et al. 2003; Mello-Thoms et al. 2012; Van der Gijp et al. 2014). These latter two processes are not necessarily represented in the scroll data. A second explanation could be that spending a larger percentage of time on question-relevant areas can have multiple underlying reasons that result in a different effect on the relation between the percentage of time spent on question-relevant areas and diagnostic accuracy. Specifically, the expected relation is that spending more time on question-relevant areas is related to a higher level of expertise (outcome Study Part 1) and therefore leads to more accurate diagnoses. Another way of reasoning could be that participants who directly recognize the correct diagnosis do not need to spend much time on the question-relevant area, and may even spend some more time on other areas to exclude other possibilities or to look for findings that support their diagnosis. A third explanation could be that residents also use non-image related information, such as the clinical information given in the question or the answer possibilities in the case of multiple choice questions, to end up with the correct diagnosis.

Although residents’ global search significantly decreased and the time spent on question-relevant significantly increased over training years (Study part 1), we did not find a link with residents’ diagnostic accuracy (Study part 2). Due to this missing link and the large observed variances in scroll measures, we conclude that the explored scroll measures cannot yet be used to monitor and evaluate expertise development in radiology residency training. An explanation for the limited direct relation between residents’ scroll measures and residents’ accuracy could be that a correct perception of the image, which is reflected in scroll measures, is necessary but not sufficient in order to end up with the correct diagnosis; spotting an abnormality does not imply that one can provide the correct diagnosis. In future studies, it would be interesting to investigate which cognitive processes are connected to specific scroll measures. For example by combining scroll data with think-aloud data, one could align residents’ thoughts during question-relevant versus question-irrelevant areas.

The current study has some limitations. Whereas the dataset enabled us to examine expertise development in scroll data of volumetric images collected longitudinally, it also limited our possibilities of statistical analyses because aggregation at the DRPT level was required. On the one hand, aggregation on the DRPT level can be viewed as a limitation to our study. As we could not distinguish between effects of different question formats nor radiological subdomain on the dependent variables in the statistical analyses. In addition, we needed to exclude marker questions in the second part of the current study, since the relation between the percentage of time spent on question-relevant areas and diagnostic accuracy differed between marker questions and other question formats. Especially in the case of marker questions, residents’ time on question-relevant areas had probably been a good predictor: The cross-sectional scatterplots showed that having spent a longer time on question-relevant areas was most often related to the provision of a correct answer. An explanation for this might be that, in order to position the marker correctly, residents necessarily needed to spend quite some time in the question-relevant area. On the other hand, aggregation on the DRPT level can be viewed as a realistic consequence of the availability of process data in practice: It is practically impossible to have all residents make the same tests in a summative testing situation if those residents do not start their training at the same time, as it is likely that residents share test items. As such, the current analyses were the best possible option under these (authentic) circumstances.

Another limitation is that we do not know what residents were doing while answering CT-scan questions. Specifically, we start logging the time as soon as the CT-scan question was displayed on the screen. It might be that residents did not look at the CT-scan for some seconds although displayed, as they were reading or answering the question, making notes or were distracted by their surroundings. Such activities could have happened at any time during image interpretation, which is why we do not believe that the data was biased in a specific direction.

The current study used scroll-data of CT-scan questions collected during semi-annual progress tests. As the test situation differs from radiology practice to some extent, results might not be completely generalisable to clinical practice. For example, in multiple choice questions residents could have provided the correct diagnosis by chance, which is impossible in clinical practice. More specific, the possibility to provide a correct diagnosis by chance may have affected the data to some extent: a resident who spent more percentage of time on question-irrelevant areas could still have provided the correct diagnosis, making it a worse predictor for diagnostic accuracy.

In conclusion, this study revealed that radiology residents’ scroll patterns align with expertise theories: percentage of time spent on full runs (i.e., global search) decreased with experience and percentage of time spent on question-relevant areas increased with experience. However, the same scroll patterns could not be used as objective predictors of residents’ diagnostic accuracy. Therefore, the relation between scroll patterns and performance needs to be further examined, before insights gained from scroll patterns can be of added value for the optimisation of radiology training programs.

## Figures and Tables

**Fig. 6:**
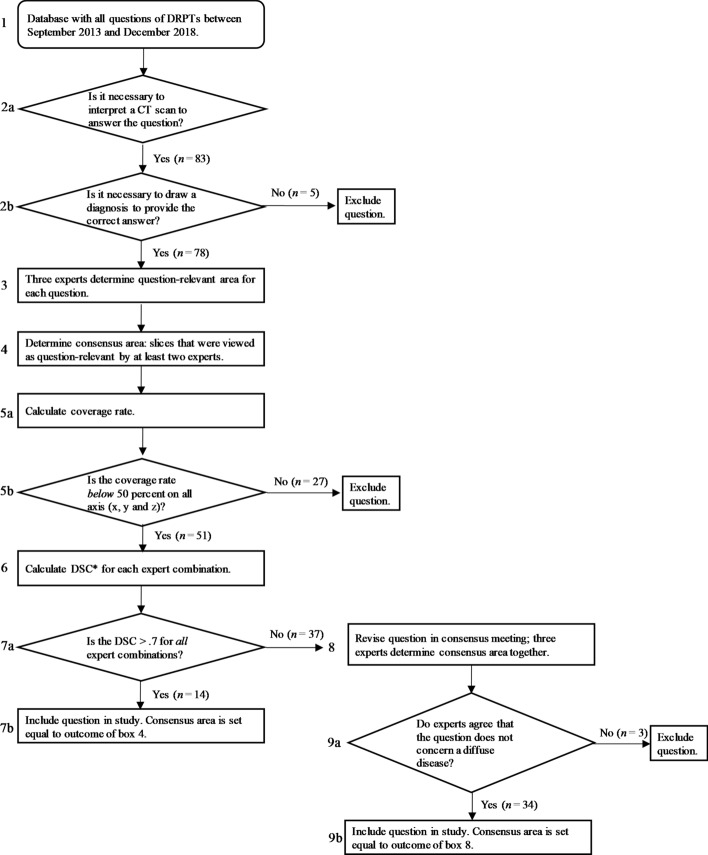
Overview of question-selection process. *DSC = Dice similarity coefficient

**Fig. 7:**
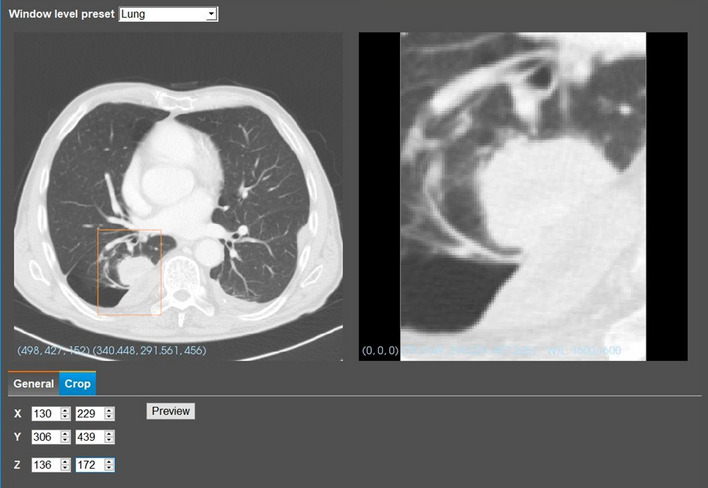
Example of a crop box indicating a question-relevant CT-scan area in x-, y- and z-direction

## Appendix 1: Selection of CT-scan questions

An overview of the steps taken to select the CT-scan image questions analysed in the current study is displayed in Fig. 6. Furthermore, the next paragraphs provide a detailed step-by-step explanation aligned with the numbering in the overview.

First, we checked in the database with all questions of DRPTs between September 2013 and April 2018, which questions were image-questions that needed the interpretation of a single CT scan. Although MRI series can also provide information of *volumetric* image interpretation, this might differ from CT-scans. We focused on CT scans, as there were only a few MRI series in the dataset. In addition, some questions needed the interpretation of *two* CT scans, since logfiles did not discriminate between CT scans *within* a question we were not able to determine which CT scan residents were viewing. Therefore, we only included questions with a *single* CT scan. All other questions were immediately excluded.

Second, for each CT-scan question we determined if it was necessary to provide a diagnosis in order to give to correct answer. Only questions for which it was necessary to make a diagnosis were included. For example, questions that only asked to mark a certain anatomical structure were excluded (e.g., “mark the tip of the Appendix”).

Third, three experts (i.e., three radiologists) individually indicated the question-relevant CT-scan area for each question, via a crop box that provided a minimum and maximum slice number in x-, y- and z-direction (see Fig. 7. In case residents were allowed to scroll in x-, y- and z-direction (i.e., sagittal, coronal and axial; 53 out of 78 questions), the crop box provided boundaries for all three axis. However, in case residents were *only* allowed to scroll in z-direction (i.e., axial direction; 25 out of 78 questions), the crop box indicated boundaries on the z-axis, whereas the x- and y-axis were fixed to their full width.

Fourth, we determined a consensus area, defined by the slices that were selected as question relevant by at least two experts (i.e., the median of each three boundary values either on all three axis or *only* on the z-axis).

Fifth, we calculated the coverage rate of the consensus area in x-, y- and z-direction, by dividing the number of question-relevant slices on *one* axis by the total amount of slices present on that axis. For example, if experts reached consensus over 86 slices that deemed to be question-relevant in x-direction, in a CT-scan that contained 512 slices in x-direction, the coverage rate on x was 16.8%. Logically, if x- and y-direction were fixed coverage rate was *only* calculated in z-direction. Questions were immediately excluded from the study if the coverage rate of the consensus area was above 50 percent in at least *one* direction.

Sixth, for the remaining questions, we calculated Dice Similarity Coefficient (DSC) between all expert combinations as a measure of interrater agreement. The DSC is a spatial overlap index ranging from 0, indicating no spatial overlap between two volumes, to 1, indicating complete overlap (cf., Cuingnet et al. 2012; Shahzad et al. 2013; Wolz et al. 2012). The DSC is obtained by dividing two times the consensus volume of two experts by the sum of the two volumes indicated by expert 1 and expert 2 (see Eq. 2).

2 $$DSC = \frac{{2\left| {{\text{V}}_{\rm exp1} \cap {\text{V}}_{\rm exp2}} \right| }}{{\left| {{\text{V}}_{\rm exp1}} \right| + \left| {{\text{V}}_{\rm exp2}} \right|}}$$

To provide an example of the calculation of the DSC for *one* expert combination in *one* CT-scan question, we use the boundaries of the question-relevant crop boxes displayed in Table 7. To determine the question-relevant volume of each expert (i.e., denominator of Eq. 2), we need a scale; therefore slices were converted to millimetres using the question-specific CT-scan characteristics. In this example question, *one* slice in x was equal to 0.36 mm, in y to 0.36 mm and in z to 0.40 mm. Therefore, the question-relevant crop box of expert 1 had a volume of 45,147.94 mm^3^ (65.95 mm * 47.70 mm * 14.80 mm) and of expert 2 of 13,483.37 mm^3^ (31.07 mm * 28.54 mm * 15.20 mm). The denominator, is determined by the question-relevant volume on which experts reached consensus. See Table 7 for the boundaries of the question-relevant crop on which expert reached consensus. This consensus crop yields a volume of 11,161.28 mm^3^ (31.07 mm * 25.65 mm * 14.00 mm), using the same conversion values as before. This results in a DSC of .38 (Eq. 2). This indicates a low spatial overlap between experts.

**Table 7:** Example of question-relevant slices indicated by two experts

	Xmin	Xmax	Ymin	Ymax	Zmin	Zmax
Expert 1	115	292	104	236	240	277
Expert 2	154	240	96	175	237	275
Consensus expert 1 and 2	154	240	104	175	240	275

Seventh, questions with a DSC higher than .7 for *all* expert combinations were immediately included in the current study. A value of .7 was chosen as this indicates a moderate spatial overlap between experts, based on previous studies that measured spatial accuracy in CT-scans using DSC (Shahzad et al. 2013; Wolz et al. 2012). Specifically, since the question-relevant area was set equal to the consensus area which was—as stated before—defined by the slices that were selected as question-relevant by at least two experts, moderate overlap per expert combination was viewed as acceptable.

Eight, questions with a DSC lower than .7 for at least *one* expert combination were revised in a consensus meeting with all three experts. The number of questions that needed revision was quite high (*n* = 38), suggesting the need for a shared definition of the term ‘question-relevant area’. Therefore, experts formulated a shared definition at the start of the consensus meeting: “A selection of slices that are most relevant for the provision of the correct diagnosis; for an area to be deemed relevant, the abnormality needs to be visible on that area”. Subsequently, all three experts simultaneously looked at the CT-scans of the to be revised questions and determined the question-relevant crop box together using the definition as ground rule.

Ninth, for revised questions the question-relevant area was set equal to the crop box determined in the consensus meeting. Revised questions were *only* included in the current study if the coverage rate of the new consensus area was *below* 50 percent in *all* directions and if experts reach agreement on inclusion.

In sum, 48 CT-scan questions were selected with an average of 5 questions per DRPT (min = 2, max = 9).

## Appendix 2: Comparison results of all tested multilevel models per outcome variable, used to determine the best fitting models

### Study part 1: Outcome variables PercTimeFullRunsAvg and PercTimeRelAreaAvg

#### Average time spent on full runs per DRPT

As described in the method section, in order to test H1a, we ran multiple successive two-level longitudinal regression models with the dependent variable *PercTimeFullRunsAvg*. Step by step we extended the intercept only model that included a fixed effect for *TrTime* and random intercepts on the resident and the DRPT level (Model 1), with a fixed effect for *DRPTdiff* (Model 2) and with a random slope for *TrTime* (Model 3).

For the two-level longitudinal model with the dependent variable *PercTimeFullRunsAvg*, a model that included fixed effects for *TrTime* and *DRPTdiff* as well as random intercepts on DRPT and resident level (Model 2) was the best fitting model. Specifically, Model 2 had a significantly better fit with the data than a model that only included a fixed effect for *TrTime* and random intercepts on the DRPT and the resident level (Model 1), ∆χ^2^(1) = 7.68, *p *= .006. In addition, Model 3, which was Model 2 plus a random slope for *TrTime*, did not fit significantly better than Model 2, ∆χ^2^(2) = 4.16, *p *= .12. Furthermore, the random slope for *TrTime* in Model 3 was not significant, χ^2^(539)(See footnote 1) = 585.7, *p *=.08. This indicates that there were no significant individual differences in development of time spent on full runs (i.e., averaged per DRPT) over *TrTime*. In other words, no large individual differences in development rate were found. This also favoured the less complex Model 2, of which detailed results are described in the results section.

#### Average time spent on question-relevant areas per DRPT

As described in the method section, in order to test H1b, we ran multiple successive two-level longitudinal regression models with the dependent variable *PercTimeRelAreaAvg*. This was done in a similar manner as the successive models for H1a, with the outcome variable *PercTimFullRunsAvg*.

For the two-level longitudinal model with the dependent variable *PercTimeRelAreaAvg*, a model that included fixed effects for *TrTime* and *DRPTdiff* as well as random intercepts on the DRPT and the resident level (Model 2), was the best fitting model. Specifically, Model 2 had a significant better fit with the data than a model that only included a fixed effect for *TrTime* and random intercepts on the DRPT and the resident level, ∆χ^2^(1) = 331.62, *p *< .001. In addition, Model 3, which was Model 2 plus a random slope for *TrTime*, did not fit significantly better than Model 2, ∆χ^2^(2) = 5.95, *p *= .05. Furthermore, the random slope for *TrTime* in Model 3 was not significant χ^2^(539)^1^ = 2.11, *p *=.13, indicating that there were no significant individual differences in development of time spent on question-relevant areas (i.e., averaged per DRPT) over *TrTime*. In other words, no large individual differences in development rate were found. This also favoured the less complex Model 2, of which detailed results are described in the results section.

### Study part 2: outcome variable diagnostic accuracy

#### Diagnostic accuracy aggregated on the DRPT level

As described in the method section, in order to test H2a and H2b, we ran multiple successive two-level longitudinal logistic regression models with the proportional outcome variable number of questions correct per number of (for the current study selected) questions in a DRPT. Step by step we extended the intercept only model that included a fixed effect for *TrTime* and a random intercept on the DRPT level (Model 1), with fixed effects for *DRPTdiff, PercTimeFullRunsAvg *and *PercTimeRelAreaAvg* (Model 2), and with a random slope for *TrTime* (Model 3).

A two-level longitudinal logistic regression model that included fixed effects for *TrTime*, *DRPTdiff*, *PercTimeFullRunsAvg* and *PercTimeRelAreaAvg* as well as a random intercept on the DRPT level, was the best fitting model that included all variables of interest. Specifically, Model 2 had a significant better fit with the data than Model 1, a model that only included a fixed effect for *TrTime* and a random intercept on the DRPT level, ∆χ^2^(3) = 9854.62, *p *< .001. Finally, in Model 3, which was Model 2 plus a random slope for *TrTime*, the random slope for *TrTime* was not significant *z* = 0.24, *p *=.81. This indicates that there were no significant individual differences in development of the number of questions correct per number of questions in a DRPT. In other words, no large individual differences in development rate were found. This favoured the less complex Model 2, of which detailed results are described in the results section.

## Appendix 3: Model equations of best fitting models

### Best fitting models study part 1

#### Outcome variable percentage of time spent on full runs averaged over DRPTs (*PercTimeFullRunsAvg*)

The model displayed below, is the best fitting model described in the result section (Model 2), which included fixed effects for *TrTime* and *DRPTdiff*, and random intercepts on the resident and the DRPT level.

Level-1 Model

$${\text{PercTimeFullRunsAvg}}_{\text{ti}} = \pi_{0i} + \pi_{1i} TrTime_{ti} + \pi_{2i} DRPTdiff_{ti} + e_{ti}$$

Level-2 Model

$$\pi_{0i} = \beta_{00} + \mu_{0i}$$

$$\pi_{1i} = \beta_{10}$$

$$\pi_{2i} = \beta_{20}$$

Mixed Model

$${\text{PercTimeFullRunsAvg}}_{\text{ti}} = \beta_{00} + \beta_{10} TrTime_{ti} + \beta_{20} DRPTdiff_{ti} + e_{ti} + \mu_{0i}$$

### Outcome variable percentage of time spent on questions relevant areas averaged over DRPTs (*PercTimeRelAreaAvg*)

The model displayed below, is the best fitting model described in the result section (Model 2), which included fixed effects for *TrTime* and *DRPTdiff*, and random intercepts on the resident and the DRPT level.

Level-1 Model

$${\text{PercTimeRelAreaAvg}}_{\text{ti}} = \pi_{0i} + \pi_{1i} TrTime_{ti} + \pi_{2i} DRPTdiff_{ti} + e_{ti}$$

Level-2 Model

$$\pi_{0i} = \beta_{00} + \mu_{0i}$$

$$\pi_{1i} = \beta_{10}$$

$$\pi_{2i} = \beta_{20}$$

Mixed Model

$${\text{PercTimeRelAreaAvg}}_{\text{ti}} = \beta_{00} + \beta_{10} TrTime_{ti} + \beta_{20} DRPTdiff_{ti} + e_{ti} + \mu_{0i}$$

### Best fitting model study part 2

#### Outcome variable number of questions correct per number of (for the current study selected) questions in a DRPT (*DiagAcc*)

The model displayed below is the best fitting model described in the results section (Model 2), which includes fixed effects for *TrTime*, *DRPTdiff*, *PercTimeFullRunsAvg* and *PercTimeRelAreaAvg* as well as a random intercept on the DRPT level. Furthermore a logit link function is used on the first level in order to deal with the proportional outcome variable.

Level-1 model

- E(NumberQuestionsCorrect_ti_ = 1|π_i_) = ϕ_ti_* NumberOfQuestions_ti_
- log[ϕ_ti_/(1 − ϕ_ti_)] = η_ti_
- η_ti_ = π_0i_ + π_1i_*(TrTime_ti_) + π_2i_*(DRPTdiff_ti_) + π_3i_*(PercTimeFullunsAvg_ti_) + π_4i_*(PercTimeRelAreaAvg_ti_)

Level-2 Model

- π_0i_ = β_00_ + µ_0i_
- π_1i_ = β_10_
- π_2i_ = β_20_
- π_3i_ = β_30_
- π_4i_ = β_40_

Mixed model

- η_ti_ = β_00_ + β_10_TrTime_ti_ + β_20_DRPTdiff_ti_ + β_30_PercTimeFullunsAvg_ti_
- **+** β_40_PercTimeRelAreaAvg_ti_ + µ_0i_.
